# Increased Cytotoxicity of Bimetallic Ultrasmall Silver–Platinum Nanoparticles (2 nm) on Cells and Bacteria in Comparison to Silver Nanoparticles of the Same Size

**DOI:** 10.3390/ma17153702

**Published:** 2024-07-26

**Authors:** Natalie Wolff, Nataniel Białas, Kateryna Loza, Marc Heggen, Torsten Schaller, Felix Niemeyer, Claudia Weidenthaler, Christine Beuck, Peter Bayer, Oleg Prymak, Cristiano L. P. Oliveira, Matthias Epple

**Affiliations:** 1 Inorganic Chemistry and Centre for Nanointegration Duisburg-Essen (CENIDE), University of Duisburg-Essen, Universitaetsstr. 5-7, 45117 Essen, Germany; natalie.wolff@uni-due.de (N.W.); nataniel.bialas@uni-due.de (N.B.); kateryna.loza@uni-due.de (K.L.); oleg.prymak@uni-due.de (O.P.); 2 Ernst Ruska Centre for Microscopy and Spectroscopy with Electrons, Forschungszentrum Jülich, 52428 Jülich, Germany; m.heggen@fz-juelich.de; 3 Organic Chemistry, University of Duisburg-Essen, Universitaetsstr. 5-7, 45117 Essen, Germany; torsten.schaller@uni-due.de (T.S.); felix.niemeyer@uni-due.de (F.N.); 4 Max-Planck-Institut für Kohlenforschung, 45470 Mülheim an der Ruhr, Germany; weidenthaler@mpi-muelheim.mpg.de; 5 Institute of Biology and Center for Medical Biotechnology (ZMB), University of Duisburg-Essen, Universitaetsstr. 5-7, 45117 Essen, Germany; christine.beuck@uni-due.de (C.B.); peter.bayer@uni-due.de (P.B.); 6 Institute of Physics, University of São Paulo, São Paulo 05508-090, Brazil; crislpo@if.usp.br

**Keywords:** nanoparticles, silver, gold, platinum, cytotoxicity, antibacterial effects

## Abstract

Ultrasmall nanoparticles (diameter 2 nm) of silver, platinum, and bimetallic nanoparticles (molar ratio of Ag:Pt 0:100; 20:80; 50:50; 70:30; 100:0), stabilized by the thiolated ligand glutathione, were prepared and characterized by transmission electron microscopy, differential centrifugal sedimentation, X-ray photoelectron spectroscopy, small-angle X-ray scattering, X-ray powder diffraction, and NMR spectroscopy in aqueous dispersion. Gold nanoparticles of the same size were prepared as control. The particles were fluorescently labeled by conjugation of the dye AlexaFluor-647 via copper-catalyzed azide-alkyne cycloaddition after converting amine groups of glutathione into azide groups. All nanoparticles were well taken up by HeLa cells. The cytotoxicity was assessed with an MTT test on HeLa cells and minimal inhibitory concentration (MIC) tests on the bacteria *Escherichia coli* and *Staphylococcus xylosus*. Notably, bimetallic AgPt nanoparticles had a higher cytotoxicity against cells and bacteria than monometallic silver nanoparticles or a physical mixture of silver and platinum nanoparticles. However, the measured release of silver ions from monometallic and bimetallic silver nanoparticles in water was very low despite the ultrasmall size and the associated high specific surface area. This is probably due to the surface protection by a dense layer of thiolated ligand glutathione. Thus, the enhanced cytotoxicity of bimetallic AgPt nanoparticles is caused by the biological environment in cell culture media, together with a polarization of silver by platinum.

## 1. Introduction

Nanoparticles are the basis of nanomedicine, i.e., the targeted delivery of drugs by therapeutic or theranostic nanoparticles [1,2,3,4]. Imaging is also possible with the application of suitably functionalized nanoparticles [5,6,7]. Another important area is the fight against pathogenic bacteria, e.g., during implant-associated infections. Here, nanoparticles with bactericidal properties represent a promising alternative to antibiotics [8,9]. One approach is to use nanoparticles as carriers of drugs against bacteria, and an alternative is the controlled release of bactericidal ions like silver or copper to exert a specific bactericidal action.

Silver nanoparticles have gained some prominence in this field due to their well-known antibacterial effect [10,11,12,13]. However, it has been demonstrated that silver ions are toxic against eukaryotic cells (e.g., healthy tissue) as well [14]. An alternative approach is to prepare bimetallic nanoparticles where silver is mixed with a nobler metal like gold [15,16,17,18,19,20] or platinum [21,22,23]. Another option is to make the nanoparticles very small so that they are more mobile in the body and less prone to form a protein corona on their surface [24].

Here, we present the biological effects of ultrasmall bimetallic silver–platinum nanoparticles with variable metal ratio (diameter about 2 nm), coated with glutathione (GSH), and compare their bactericidal effect with the cytotoxic effect against eukaryotic cells. In addition, monometallic nanoparticles of silver, platinum, and gold (as control) of the same size were studied, as well as a physical mixture of silver and platinum nanoparticles.

## 2. Materials and Methods

### 2.1. Chemicals and Reagents

Ultrapure water prepared with a Purelab ultra instrument (ELGA, Celle, Germany) with a specific resistivity of 18.2 MΩ was used for all syntheses and analyses, unless otherwise noted. All glassware for the syntheses was cleaned with boiling *aqua regia* and rinsed twice with water before use.

As metal precursors, silver nitrate (AgNO_3_, 99%, Carl Roth, Karlsruhe, Germany), hexachloridoplatinum(+IV) acid (H_2_PtCl_6_, 8 wt% in H_2_O; Sigma-Aldrich, Steinheim, Germany), and tetrachloridoauric(+III) acid (HAuCl_4_; prepared by dissolution of elemental gold in *aqua regia*) were used. Sodium borohydride (NaBH_4_, 96%), L-glutathione (GSH, 98%), copper(II)sulfate pentahydrate (>99%), sodium L-ascorbate (>99%), and Spin-X^®^ UF 30 kDa MWCO PES spin filters (Corning^®^) were obtained from Sigma-Aldrich. Sodium hydroxide (NaOH, 1 M), hydrochloric acid (HCl, 37%), and nitric acid (HNO_3_, 67%) were obtained from Bernd Kraft (Duisburg, Germany). Methanol (99.8%) was obtained from FisherScientific (Geel, Belgium). Aminoguanidine hydrogen carbonate (98%) was obtained from Alfa Aesar (Kandel, Germany). Tris(3-hydroxypropyltriazolylmethyl)amine (THPTA, >95%) and AlexaFluor-647-alkyne (95%) were obtained from Jena Bioscience (Jena, Germany). Dodecane (99%) and 2-(4,5-dimethylthiazol-2-yl)-3,5-diphenyl-2H-tetrazol-3-ium bromide (MTT) were obtained from ThermoFisher Scientific (Schwerte, Germany). Dimethyl sulfoxide (DMSO, >99.5%) was obtained from Carl Roth. For differential centrifugal sedimentation (DCS), a PVC nanoparticle calibration dispersion (Lot#149, 1.385 g L^−1^) was obtained from CPS Instruments Inc. (Oosterhout, The Netherlands). Deuterium oxide (D_2_O, 99.9%) was obtained from Deutero GmbH (Kastellaun, Germany). 10 kDa spin filters were obtained from Merck (Darmstadt, Germany). Imidazole-1-sulfonyl azide hydrogen sulfate was prepared as reported earlier [25].

### 2.2. Electron Microscopy

High-resolution transmission electron microscopy (HRTEM) was carried out with an FEI Titan transmission electron microscope (ThermoFisher, Schwerte, Germany, which was aberration-corrected with a Cs-probe corrector (CEOS Company, Heidelberg, Germany) and operated at an accelerating voltage of 300 kV [26]. The nanoparticles were dispersed in water, followed by placing a drop of the suspension onto a carbon-coated copper grid and allowing it to dry under ambient conditions. The core diameter was determined manually by measuring the size of at least 100 particles from different HRTEM images.

### 2.3. X-ray Powder Diffraction (XRD)

X-ray powder diffraction (XRD) was performed with a Bruker D8 Advance diffractometer (Bruker Company, Billerica, MA, USA), with Cu Kα radiation (*λ* = 1.54 Å) operating at 40 kV and 40 mA. A dispersion of nanoparticles was placed on a silicon single-crystal sample holder to minimize scattering and dried in air. The samples were measured in reflection mode from 20 to 90° 2Θ with a step size of 0.02° and a counting time of 8 s per step. This resulted in a total measurement time of 500 min. Qualitative phase analysis was performed with Diffrac.Suite EVA V1.2 from Bruker with the patterns of the metals Ag (#04-0783) and Pt (#04-0802) and their oxides Ag_2_O (#41-1104) and PtO (#47-1171) from the ICDD database.

### 2.4. Small-Angle X-ray Scattering (SAXS)

Small-angle X-ray scattering was performed at the EMUSAXS center of the Institute of Physics, University of São Paulo. The laboratory-based equipment Xeuss 2.0 was used for the data acquisition. The source was a Genix3D Cu Kα X-ray tube (*λ* = 1.5418 Å), equipped with a Focus3D mirror. The beam was collimated with two sets of scatterless slits, leading to a beam size of 0.7 × 0.7 mm^2^. The 2D scattering images were collected on a Dectris-Pilatus 300k detector placed at a 537 mm distance from the sample. The images were integrated with the program Fit2D [27]. 1D curves of the intensity as function of the reciprocal space momentum transfer modulus *q* were obtained. *q* is defined as *q* = 4π sin(*θ*)/*λ*, where *θ* is the scattering angle. The liquid samples (nanoparticles dispersed in water) were placed on reusable sample holders composed of borosilicate glass capillaries (Hilgenberg, Germany) of 1.5 mm diameter, glued on stainless steel cases. The holder was closed with rubber caps which allowed the measurements in vacuum. In this way, samples and blank were measured under exactly the same conditions, which is crucial for the proper data treatment. The data treatment was performed with the program SuperSAXS [28] using plain water as blank. The scattering from water was also used for absolute scale normalization.

The scattering data were analyzed by a polydisperse hard spheres model, as already used in previous work with nanoparticles [29]. In this model, the particles are described with a central radius *R*, a polydispersity *σ*, a hard spheres interaction radius *R*_HS_, and a volume fraction *η*.

### 2.5. NMR Spectroscopy

Proton and carbon nuclear magnetic resonance (NMR) analyses were conducted with a Bruker AVIII HD spectrometer (Bruker Company, Billerica, MA, USA). The spectrometer was equipped with a nitrogen-cooled probe and operated at 600.13 MHz for ^1^H and 150.90 MHz for ^13^C. For ^1^H NMR acquisition, the excitation sculpting technique to attenuate the solvent’s signal was used. Similarly, water signal pre-saturation was employed during COSY, HSQC, and HMBC experiments to achieve analogous suppression.

### 2.6. DOSY-NMR Spectroscopy

DOSY-NMR spectroscopy was performed and analyzed as previously described [30] on a Bruker Avance III 700 MHz spectrometer (Bruker Company, Billerica, MA, USA) equipped with a 5 mm TCI cryoprobe with a *z*-gradient at 25 °C. ^1^H DOSY spectra were recorded with a diffusion time of *Δ* = 100 ms, a pulsed gradient duration of *δ* = 3.5 ms, and the gradient strength ranging from 5–95% of the maximum (66 G cm^−1^) in 32 steps. Spectra were processed with Topspin 3.5 (Bruker). The linearized diffusion data were analyzed according to the Stejskal–Tanner equation [31,32]:

(1) lnII0=−γ2·δ2·Δ −δ3·D·G2

with *I* the signal intensity, *I*_0_ the signal intensity without gradient, *γ* the gyromagnetic ratio of ^1^H, *δ* the diffusion gradient pulse length, *Δ* the diffusion delay, *G* the gradient strength, and *D* the translational diffusion coefficient.

The Stejskal–Tanner plots for three GSH signals (H1/H6 3.8 ppm, H4 2.6 ppm, 2.2 ppm) were first analyzed separately. Then, the data points of all three signals were averaged, with the error bars representing their standard deviation. While the intrinsic standard error of the Stejskal–Tanner fit is small (<2%), we estimate the error of the diffusion coefficient to about 20% due to the manual integration and potential overlaying of small signals from impurities.

The hydrodynamic diameter was calculated according to the Stokes–Einstein equation [33]:

(2) $$d_{H}=\frac{k_{B}\cdot T}{3\cdot \pi \cdot \eta \cdot D}$$

with *d*_H_ the hydrodynamic diameter, k_B_ the Boltzmann constant, *T* the temperature in K, *η* the dynamic viscosity at 25 °C, and *D* the translational diffusion coefficient.

### 2.7. X-ray Photoelectron Spectroscopy (XPS)

X-ray photoelectron spectroscopy (XPS) was performed with a SPECS spectrometer (SPECS Surface Nano Analysis GmbH, Berlin, Germany), which was equipped with a Phoibos 150 1D-DLD hemispherical energy analyzer. The monochromatized Al Kα X-ray source (*E* = 1486.6 eV) was operated at 15 kV and 200 W. The pass energy was set to 20 eV for high-resolution scans, and the medium area mode was used as lens mode. The base pressure in the analysis chamber was set to 1·10^−10^ mbar during the experiment. All spectra were referred to C 1s at 284.5 eV to account for charging effects. The spectra were evaluated with the CasaXPS software v2.3.26rev1.1s [34].

### 2.8. Elemental Analysis (AAS, ICP-MS)

Atomic absorption spectroscopy (AAS) with a Thermo Electron M-series spectrometer (ThermoFisher, Schwerte, Germany) graphite tube furnace, operated in accordance with DIN EN ISO/IEC 17025:2005), was used to determined the concentrations of Ag, Au, and Pt concentrations in the nanoparticle dispersions. 10 µL of a nanoparticle dispersion was dissolved in concentrated nitric acid (955 µL) and diluted with 3 mL water to determine Ag. 10 µL of nanoparticle dispersion was dissolved in *aqua regia* (950 µL) and diluted with 3 mL water to determine Au and Pt. The concentrations of Ag, Au, Pt, and S were determined by inductively coupled plasma mass spectrometry (Spectro model Spectro Arcos after microwave digestion) at Mikroanalytik-Labor Kolbe (Oberhausen, Germany).

### 2.9. Differential Centrifugal Sedimentation (DCS)

Differential centrifugal sedimentation, also known as analytical disc centrifugation, was performed with a CPS Instruments DC 24000 disc centrifuge (24,000 rpm, 29,000 relative centrifugal force; rcf). Different sucrose solutions (8 and 24 wt%) were used to obtain a density gradient. Dodecane (0.5 mL) was added as a top layer to prevent evaporation. Before each measurement, a dispersion of polyvinylchloride (PVC) latex in water with a specific hydrodynamic diameter of 483 nm by CPS was used for calibration. A volume of 100 µL was added to the nanoparticle dispersion. For the calculation of the hydrodynamic diameter of the monometallic nanoparticles, the densities of elemental silver (10.49 g cm^−3^), elemental platinum (21.45 g cm^−3^), and elemental gold (19.32 g cm^−3^) were used. For the bimetallic nanoparticles, the densities of elemental silver and elemental platinum were used according to the molar ratio of the particles.

### 2.10. UV-Vis Spectroscopy

UV-Vis spectroscopy was performed with water-dispersed nanoparticles from 200 nm to 800 nm (600 µL sample volume) with a Genesis 50 instrument (ThermoFisher, Schwerte, Germany). Background correction was performed with ultrapure water as reference.

### 2.11. Fluorescence Spectroscopy

The fluorescence spectra were measured with water-dispersed nanoparticles on a Cary Eclipse spectrometer (Agilent Technologies, Santa Clara, CA, USA) in a fluorescence cuvette (600 µL sample volume).

### 2.12. Synthesis of Glutathione-Coated Nanoparticles

The syntheses of glutathione-coated monometallic gold, platinum, and silver nanoparticles and of bimetallic Ag_50_Pt_50_ nanoparticles were reported earlier [30]. The synthesis of Ag_70_Pt_30_ and Ag_20_Pt_80_ nanoparticles was carried out in the same way as with Ag_50_Pt_50_ particles by changing the ratio of silver to platinum and adjusting the reaction conditions. However, we found that the ratio of the elements found in the nanoparticles was not the same as the ratio of the elements in the precursor solution; i.e., silver was incorporated more easily than platinum into the bimetallic nanoparticles. With a series of experiments with different metal ratios, followed by elemental analysis of the bimetallic nanoparticles, the synthesis parameters were optimized.

To synthesize bimetallic Ag_20_Pt_80_ nanoparticles, 400 mL water was added to a 1 L round-bottomed flask. The water was degassed for 15 min with argon. Then, 450 µL of 100 mM silver nitrate solution (4.5 µmol, 0.5 mg Ag) and 4.1 mg glutathione (85.5 µmol) dissolved in 1 mL water were added. The solution was stirred for 30 min. Next, 445 µL H_2_PtCl_6_ (*c*(Pt) = 37.5 g L^−1^, 85.5 µmol, 16.7 mg Pt) and 26.0 mg glutathione (85.5 µmol) dissolved in 1 mL water were added and again stirred for 30 min. After that, 83.3 mg NaBH_4_ (2.2 mmol) dissolved in 2 mL ice-cold water was quickly added. The stirring was continued for 30 min. The final dispersion had a brown color. Most of the water was removed under vacuum in a rotary evaporator. The Ag_20_Pt_80_ nanoparticles were isolated by spin filtration (10 kDa Amicon spin filters at 4000 rpm, 2500× *g*, 20 min). They were then washed twice with 0.1 M NaOH and six times with water to remove unbound GSH and synthesis by-products. As determined with AAS, the yields were 90% (0.44 mg, 4.05 µmol) for silver and 21% (3.45 mg, 17.66 µmol) for platinum. With ICP-MS, the yields were 81% (0.39 mg, 3.65 µmol) for silver and 23% (3.83 mg, 19.6 µmol) for platinum. The molar ratio in the particles was 19% Ag to 81% Pt according to AAS and 16% Ag to 84% Pt according to ICP-MS.

To synthesize bimetallic Ag_70_Pt_30_ nanoparticles, 400 mL water was added to a 1 L round-bottomed flask. The water was degassed for 15 min with argon. Then, 2.25 mL of 200 mM silver nitrate solution (45 µmol, 4.85 mg Ag) and 40.5 mg glutathione (135 µmol) dissolved in 1 mL water were added. The solution was stirred for 30 min. Next, 234 µL H_2_PtCl_6_ (*c*(Pt) = 37.5 g L^−1^, 45 µmol, 8.78 mg Pt) and 13.9 mg glutathione (45 µmol) dissolved in 1 mL water were added and again stirred for 30 min. After that, 76 mg NaBH_4_ (2.0 mmol) dissolved in 2 mL ice-cold water was quickly added. The stirring continued for 30 min. The final dispersion had a brown color. Most of the water was removed under vacuum in a rotary evaporator. The Ag_70_Pt_30_ nanoparticles were isolated by spin filtration (10 kDa Amicon spin filters at 4000 rpm, 2500× *g*, 20 min). They were then washed twice with 0.1 M NaOH and six times with water to remove unbound GSH and synthesis by-products. AAS yields were 93% (4.53 mg, 42.0 µmol) for silver and 38% (3.29 mg, 16.9 µmol) for platinum. With ICP-MS, the yields were 96% (4.67 mg, 43.3 µmol) for silver and 39% (3.42 mg, 17.5 µmol) for platinum. The molar ratio in the particles was 71% Ag to 29% Pt according to AAS and 71% Ag to 29% Pt according to ICP-MS.

### 2.13. Fluorescent Labeling of GSH-Coated Nanoparticles

The surface azidation of the mono- and bimetallic GSH-coated nanoparticles was performed according to the procedure reported by Klein et al. [25] The click reaction of azide-containing nanoparticles with alkyne-terminated dyes was performed with a modified synthesis scheme reported by Klein et al. [25] and van der Meer et al. [35].

A stock solution of Cu-THPTA was prepared by mixing 2 mL CuSO_4_ solution (5 mM, 10 µmol, 0.636 mg Cu), 2 mL saturated aminoguanidine solution, and 4 mL THPTA solution (48 mM, 192 µmol, 84 mg).

For the surface azidation of gold nanoparticles, Au-GSH nanoparticles (15 mg Au, 0.31 µmol NP) were dispersed in 18 mL water and 54 mL methanol in a 250 mL round-bottom flask for the surface azidation reaction. The azide transfer reagent imidazole-1-sulfonyl azide hydrogen sulfate (ISA; 184 mg, 676 µmol) was dissolved in 2 mL water and added to the reaction solution under vigorous stirring. Then, K_2_CO_3_ (94 mg, 676 µmol, dissolved in 2 mL water) was added to the reaction solution. The solution became cloudy and grayish after the addition of the reagents. Immediately thereafter, 1.04 mL of 5 mM CuSO_4_ solution (5.2 µmol) and 1 mL of 1 M NaOH were added. Water (about 20 mL) was then added until the turbidity had mostly disappeared. The solution was stirred at room temperature for 72 h. Prior to purification, the reaction solution was diluted to a total volume of 250 mL with water to keep the methanol content as low as possible for the subsequent spin filtration due to the sensitivity of the spin filters to organic solvents. The retrieved Au-N_3_ particles were washed twice with 0.1 M NaOH and six times with water by spin filtration (10 kDa Amicon spin filter at 4000 rpm, 2500× *g*, 20 min). The yield was 74% (11 mg Au) as determined by AAS.

For conjugation with AF647, Au-N_3_ nanoparticles (62 nmol nanoparticles, 3 mg Au) were added to a 25 mL round bottom flask in 10 mL water. The solution was stirred and 100 µL 11.2 mM AF647-alkyne (1.12 µmol, 1 mg, 18 equivalents AF647 per nanoparticle) was added to the dispersion. Then 583 µL of the Cu-THPTA stock solution was added. Then 301 µL sodium ascorbate solution (10 mM, 3.6 µmol, 0.6 mg) was added to the nanoparticle dispersion. The reaction solution was stirred for 17 h at room temperature under light exclusion. Spin filtration was used for purification. Dispersions were centrifuged in Amicon 10 kDa spin filter at 4000 rpm, 2500× *g*, for 20 min and washed with water until the filtrate was colorless (at least 12 times). The yield was 80% (2.4 mg Au) as determined by AAS.

The adapted synthesis conditions for the surface azidation and the click reaction for silver, platinum, and silver–platinum nanoparticles are summarized in Table 1 and Table 2. All other conditions and parameters were the same as described here for gold nanoparticles.

### 2.14. Uptake of M-AF647 Nanoparticles by HeLa Cells

HeLa cells (obtained from American Type Culture Collection, ATCC, Manassas, USA were cultured at 37 °C in 5% CO_2_ in Gibco^TM^ Dulbecco’s modified Eagle’s medium (DMEM), supplemented with 10% fetal bovine serum (FBS, obtained from ThermoFisher), 100 U mL^−1^ penicillin, 100 U mL^−1^ streptomycin, 1 mM Gibco^TM^ sodium pyruvate, and 1 mM Gibco^TM^ GlutaMAX. After a confluence of 70–90% was reached (after two to three days), the cells were passaged by trypsinization with 0.05% Gibco^TM^ Trypsin-EDTA. Between each step of the experiment, the cells were washed three times with Gibco^TM^ Dulbecco’s buffered saline (DPBS).

The uptake of AF647-terminated ultrasmall nanoparticles by HeLa cells was analyzed in an 8-well chamber polymer slide surface modified with ibiTreat for tissue culture applications (µ-Slide 8-well, ibidi) by multiple focal plane confocal laser scanning microscopy (CLSM; TCS SP8X Falcon instrument from Leica Microsystems with a 63×/1.2 water immersion objective). Briefly, 2·10^5^ cells were seeded per well and incubated with 0.2 mL DMEM overnight at 37 °C in 5% CO_2_ atmosphere. Finally, the cells were incubated with AF647-terminated nanoparticles at a concentration of 12.5 µg mL^−1^ (metal content) nanoparticles per well. HeLa cells cultured in medium alone served as control group. After incubation for 24 h, the cells were washed three times with DPBS and fixed with a 4 vol% formaldehyde solution, according to standard protocols. The actin cytoskeleton of the cells was stained with AlexaFluor-448 phalloidin. Cell nuclei were stained with DAPI. Excitation wavelengths were 405 nm for DAPI (emission: 420 to 460 nm), 488 nm for AlexaFluor-488 (emission: 495 to 515 nm), and 647 nm for AF647 (emission: 660 to 700 nm). For the CLSM images, *z*-stacks were taken across the cells at a distance of 200 nm. All nanoparticles were investigated after four weeks of storage in dispersion.

### 2.15. MTT Tests of M-GSH Nanoparticles with HeLa Cells

The cell viability after nanoparticle incubation was determined with an MTT assay. HeLa cells were first seeded at a density of 20,000 cells per well in a 24-well plate and then incubated with 0.5 mL DMEM overnight at 37 °C and 5% CO_2_ atmosphere. The cells were then incubated with GSH-terminated nanoparticles. The dispersions of the nanoparticles were tested at concentrations between 2.5 µg mL^−1^ and 100 µg mL^−1^ (metal content). HeLa cells cultured in medium served as control. After the cells had been incubated for 24 h, they were washed three times with DPBS to remove the nanoparticles. To prepare the staining solution, 30 mg MTT was dissolved in 5 mL DPBS and diluted with 25 mL DMEM to a final concentration of 1 mg mL^−1^. To each well, 0.3 mL of the staining solution was added, and the cells were incubated for 1 h at 37 °C and 5% CO_2_ atmosphere. The solution was removed and 0.3 mL of DMSO was added into each well and incubated for 30 min at room temperature. The amount of dissolved formazan was quantified in a 96-well plate with a Multiscan plate reader (Thermo Fisher, Schwerte, Germany) at 570 nm. For each nanoparticle type and concentration, at least two independent cell culture experiments were performed. All nanoparticles were investigated after four weeks of storage in dispersion.

### 2.16. Antibacterial Tests of M-GSH Nanoparticles with Staphylococcus xylosus and Escherichia coli

The minimal inhibitory concentration (MIC) is defined as the lowest concentration of an antimicrobial agent or drug that inhibits the growth of a given microbial strain in vitro. Bacterial strains were cultivated in Lysogeny Broth (LB, 20 g L^−1^) and Trypticase Soy Broth (TSB). TSB was prepared by mixing CASO-Bouillon (30 g L^−1^) and yeast extract (3 g L^−1^). To prepare solid media, Agar-Agar Kobe I was added to liquid media (15 g L^−1^). All reagents for media preparation were purchased from Carl Roth. The culture media were sterilized by autoclaving (LABOKLAV 25, SHP Steriltechnik, Detzel Schloß, Germany). Liquid cultures of *Staphylococcus xylosus* DSM 6179 (Schleifer and Kloos 1975) and *Escherichia coli* DH5α were grown overnight (130 rpm, 37 °C) in a MaxQ^TM^ 4000 orbital shaker (Thermo Scientific, USA). Next, log-phase liquid cultures of bacteria were prepared by inoculating (5% *v*/*v*) sterile TSB and LB culture media from overnight cultures of *S*. *xylosus* and *E*. *coli*, respectively. Bacteria were grown (180 rpm, 37 °C) until the fresh cultures had reached an optical density of 0.6 at the wavelength of 600 nm (cell density meter WPA Biowave, Thermo Fisher, Schwerte, Germany), which indicates the logarithmic growth phase of the bacterial cultures.

MIC experiments were performed in 96-well microplates (Thermo Fisher, Schwerte, Germany). Briefly, 190 µL of M-GSH nanoparticle-containing culture medium per well was mixed with 10 µL of the appropriate bacterial log-phase culture. The plate was then incubated overnight with gentle rotation in an orbital shaker (90 rpm, 37 °C). The MIC values were determined spectrophotometrically after 24 h of incubation with a HiPo MPP-96 microplate reader (Biosan, Riga, Latvia) at a wavelength of 620 nm. Each sample was prepared and measured in triplicate. As reference, media supplemented with dissolved AgNO_3_ as source of cytotoxic silver ions were investigated. The range of 0–100 µg mL^−1^ nanoparticles/salt was studied in steps of 0, 5, 10, 15, 25, 50, 75, and 100 µg metal mL^−1^. Parallel to the MIC experiments, CFU (colony-forming unit) values of the log-phase cultures were determined on agar plates after overnight incubation at 37 °C (Heratherm^TM^ Compact, Thermo Fisher, Schwerte, Germany) to determine culture viability and bacterial cell dose per well. Bacterial colonies were counted with an SC6+ digital colony counter (VWR International, Darmstadt, Germany). All nanoparticles were investigated after four weeks of storage in dispersion.

## 3. Results

The syntheses and physico-chemical characterization data of monometallic glutathione-coated gold, silver, and platinum nanoparticles, as well as Ag_50_Pt_50_ nanoparticles, were reported earlier [30]. Bimetallic nanoparticles with the compositions of Ag_70_Pt_30_ and Ag_20_Pt_80_ were prepared in addition to assess the effect of the metal ratio in the nanoalloys. All particles were synthesized by reduction of the corresponding metal salts with NaBH_4_ in a modified Brust–Schiffrin synthesis with glutathione (GSH) as stabilizing ligand [36,37,38,39]. The characterization data of the additional bimetallic compositions Ag_70_Pt_30_ and Ag_20_Pt_80_ were well in line with the other mono- and bimetallic particles.

**Figure 1:**
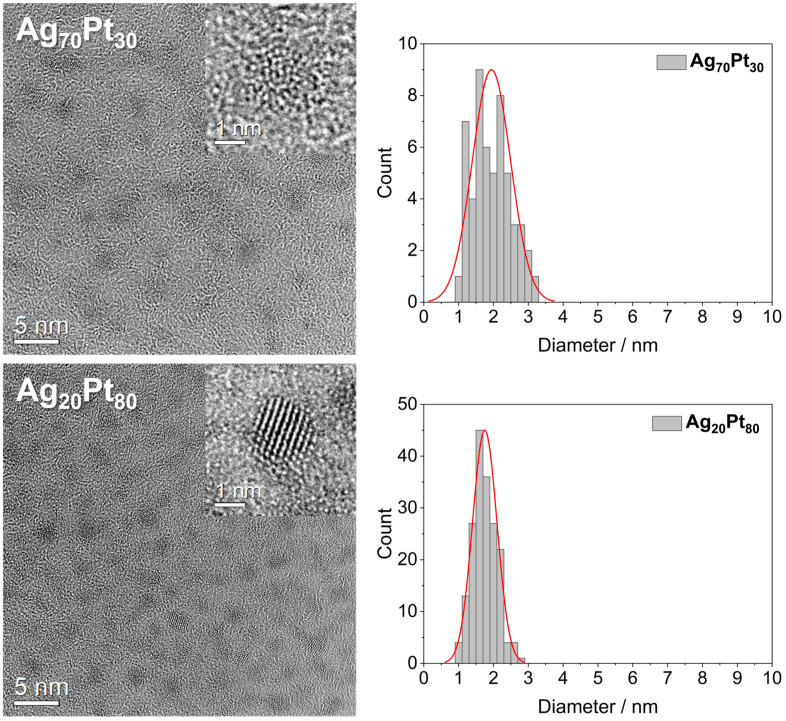
HRTEM images of GSH-coated Ag_70_Pt_30_ and Ag_20_Pt_80_ bimetallic nanoparticles, together with manually determined particle size distributions (red: fit line). A magnified particle is shown in the upper right corner of each overview image. Ag_70_Pt_30_ particles were amorphous and Ag_20_Pt_80_ particles were crystalline. Note the higher contrast of the Pt-rich nanoparticles due to the heavier Pt atoms.

HRTEM images of the GSH-coated nanoparticles are shown in Figure 1. The average particle diameter was about 2 nm. Electron diffraction accompanied by Fast Fourier Transform (FFT) indicated amorphous Ag_70_Pt_30_ and crystalline Ag_20_Pt_80_ particles in agreement with amorphous Ag_50_Pt_50_ nanoparticles [30]. The monometallic Ag, Au, and Pt nanoparticles were all crystalline, as shown earlier by electron diffraction [30].

The hydrodynamic diameter of the nanoparticles dispersed in water was determined with differential centrifugal sedimentation (DCS; Figure S1). The diameter of the dispersed Ag_70_Pt_30_ and Ag_20_Pt_80_ nanoparticles was less than 2 nm, indicating a good dispersibility in water without detectable agglomeration. This was confirmed by dynamic light scattering (DLS) that did not give a scattering signal. Note that DCS generally underestimates the particle size because the actual density of the particles is lower due to the hydrated ligand shell [40].

Small-angle X-ray scattering showed well-dispersed nanoparticles (Figure S2). From the data analysis, a diameter of 1.0 ± 0.1 nm with polydispersities of 0.4 nm and 0.5 nm for Ag_70_Pt_30_ and Ag_20_Pt_80_, respectively, was obtained. Moderate particle interactions were observed, giving a hard sphere radius of 3.5 ± 0.1 nm and a volume fraction of 0.054 ± 0.005 for Ag_70_Pt_30_ nanoparticles. The corresponding values for Ag_20_Pt_80_ nanoparticles were 4.1 ± 0.1 nm and 0.071 ± 0.006. X-ray powder diffraction indicated very small particles, as shown by the very broad diffraction peaks with indications for the presence of oxidized silver (Figure S3). X-ray photoelectron spectroscopy confirmed the oxidized nature of the particles which was also detected earlier in Ag_50_Pt_50_ nanoparticles [30]. Silver was fully oxidized, and platinum was present as a mixture of metallic platinum and oxidized platinum species (Figure S4). NMR spectroscopy, which is possible for ultrasmall nanoparticles [41], showed the firm attachment of glutathione to the nanoparticles and the absence of free (dissolved) glutathione (Figures S5–S9). The hydrodynamic diameter of the dispersed particles, together with the ligand shell, was probed with ^1^H-DOSY-NMR spectroscopy. The number of glutathione ligands on the surface of each nanoparticle was determined to be 200 to 300 with ICP-MS (see ref. [30] and Table 3 for detailed stoichiometric and analytical data).

**Figure 2:**
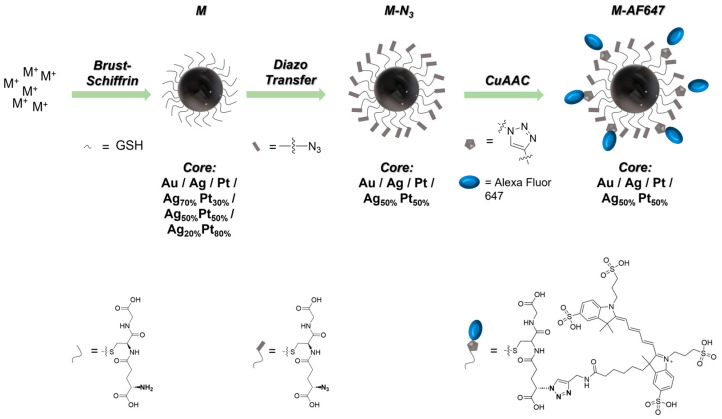
Synthetic pathway of the synthesis and the covalent attachment of AF647 molecules to the surface of ultrasmall GSH-coated nanoparticles.

Monometallic gold, silver, and platinum nanoparticles, as well as bimetallic Ag_50_Pt_50_ nanoparticles, were surface-conjugated with the fluorescent dye AlexaFluor-647 (AF647) as reported earlier for gold nanoparticles [25]. The reaction procedure is schematically depicted in Figure 2, i.e., first the synthesis of M-GSH nanoparticles, followed by surface azidation to M-N_3_ nanoparticles [25], and finally conjugation of the alkyne-terminated dye AF647 via copper-catalyzed azide-alkyne cycloaddition (CuAAC) to yield M-AF647 nanoparticles [42]. This fluorescent labeling permits us to trace the particles during cell culture experiments. The particle types Ag_70_Pt_30_ and Ag_20_Pt_80_ were not fluorescently labeled as their uptake by cells is not expected to be different from Au, Ag, Pt, and Ag_50_Pt_50_ nanoparticles (same size, just different metal cores).

With a combination of UV-Vis spectroscopy (giving the concentration of AF647) and atomic absorption spectroscopy (AAS; giving the concentration of gold nanoparticles), between 6 and 13 AF647 molecules were detected on each nanoparticle (see Table 3), in good agreement with earlier data on dye-conjugated ultrasmall gold nanoparticles [25,35,43].

**Figure 3:**
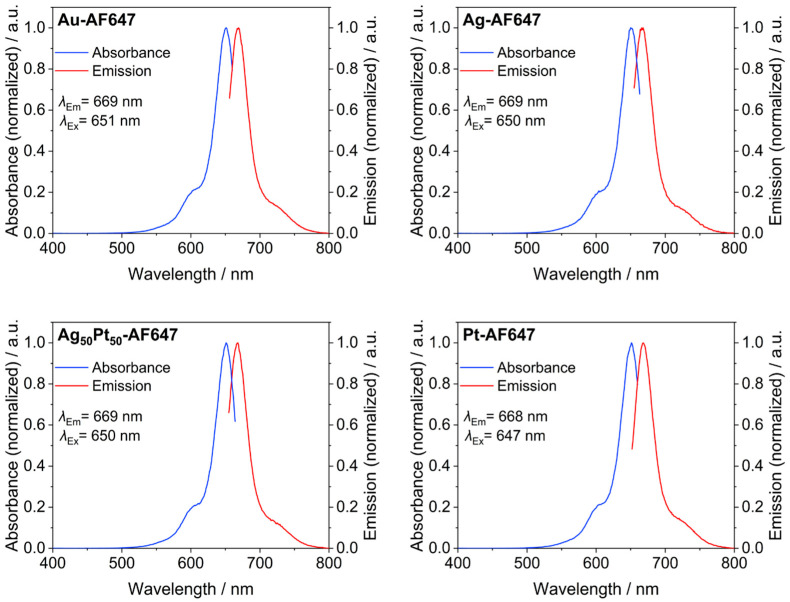
Normalized fluorescence spectra of M-AF647 nanoparticles. Absorption spectra (blue) and emission spectra (red) are shown.

Figure 3 shows fluorescence spectra of all labeled M-AF647 nanoparticles. All particles showed a distinct fluorescence, demonstrating that no significant quenching occurred as expected for ultrasmall nanoparticles [44].

**Figure 4:**
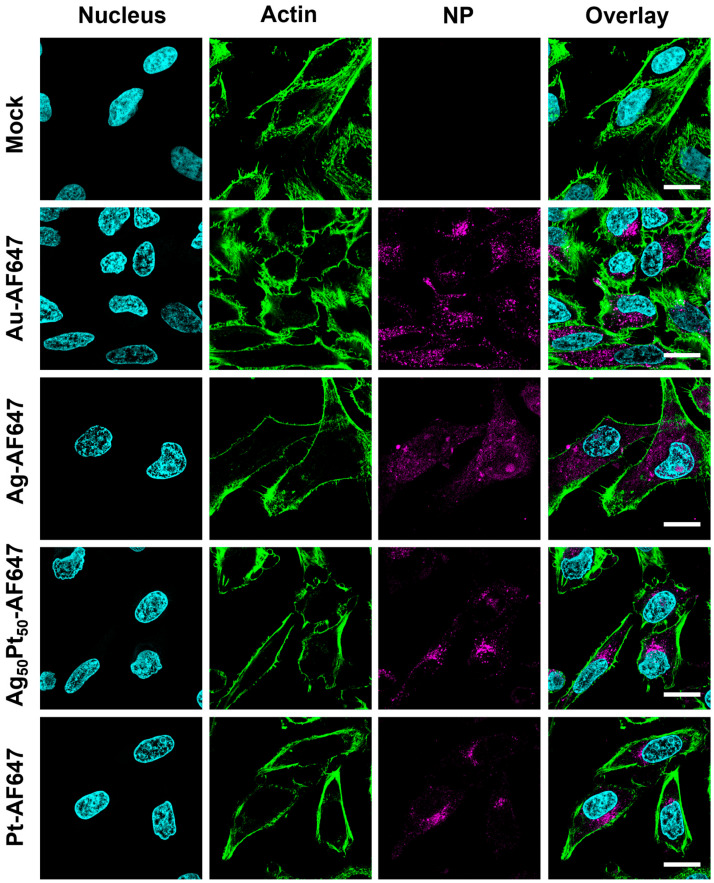
Confocal laser scanning images showing the uptake of ultrasmall AF647-functionalized nanoparticles. HeLa cells were incubated with 12.5 µg mL^−1^ nanoparticles (metal concentration) for 24 h, followed by washing, fixation, and staining. **Mock**: Untreated HeLa cells (no nanoparticles). **Nuclei**: DAPI staining (blue). **Actin**: staining with AlexaFluor™-488 phalloidin (green). **NP**: AF647 fluorescence, showing the nanoparticles (magenta). All scale bars are 20 µm.

Confocal laser scanning microscopy was used to follow the uptake of the fluorescently labeled M-AF647 nanoparticles by HeLa cells (Figure 4). All particles were well taken up by the cells.

**Figure 5:**
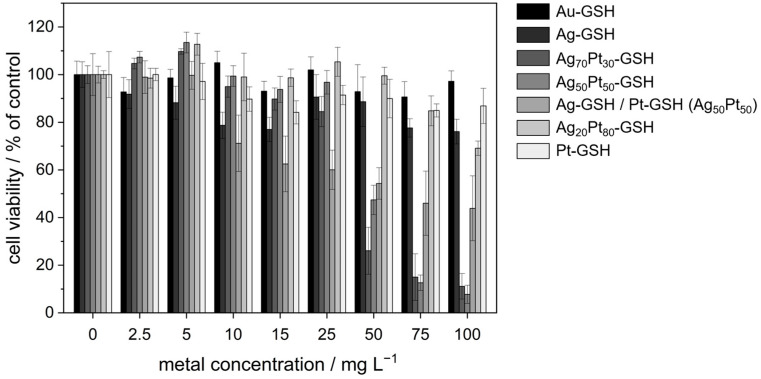
MTT viability assay carried out with HeLa cells after incubation for 24 h with water-dispersed GSH-coated nanoparticles. For the bimetallic silver nanoparticles, the given concentrations refer to the total metal concentration. Ag/Pt (50:50) represents an equimolar 50:50 physical mixture of Ag and Pt nanoparticles. The data represent the mean of two individual experiments with the error bars indicating the standard deviation (*N* = 2).

To assess the cytotoxicity of GSH-coated ultrasmall nanoparticles, an MTT assay with HeLa cells was performed (Figure 5 and Table 4). This assay provides information on the metabolic activity of the cells and serves as an indicator of cell viability and proliferation [45]. In addition to monometallic and bimetallic silver–platinum nanoparticles, a physical mixture of the monometallic nanoparticles was also used to investigate possible polarization effects. All viability data were normalized to untreated cells (100%). Cell viabilities above 100% in some cases are considered not significant and within the experimental error.

The antibacterial activity of the GSH-coated nanoparticles was assessed by determination of the minimal inhibitory concentration (MIC) in broth cultures of Gram-negative (G-) *Escherichia coli* and Gram-positive (G+) *Staphylococcus xylosus* bacteria (Table 5). MIC values of monometallic nanoparticles (Ag, Au, and Pt) were above the highest studied nanoparticle concentration (100 µg mL^−1^). In other words, the particles were not toxic for both bacterial strains. MIC values of the bimetallic nanoparticles (Ag_50_Pt_50_) ranged from 1–5 µg mL^−1^ for *S*. *xylosus* to 11–15 µg mL^−1^ for *E*. *coli*, indicating a significant cytotoxic effect against bacteria, especially towards Gram-positive *S*. *xylosus*. These concentrations are in the range that is characteristic for potent conventional antibiotics [46,47,48]. In contrast, the incubation with a physical mixture of monometallic nanoparticles (Ag/Pt 50:50) gave MIC results comparable to the monometallic nanoparticles, i.e., a low cytotoxicity.

**Figure 6:**
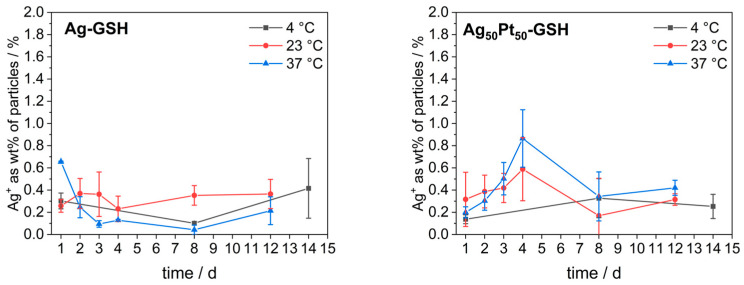
Dissolution of monometallic silver and bimetallic GSH-coated Ag_50_Pt_50_ nanoparticles stored in non-degassed pure water (i.e., in the presence of dissolved oxygen) at 4 °C, 25 °C, and 37 °C for up to 14 days.

It is now well accepted that silver nanoparticles dissolve over time under the release of cytotoxic silver ions [49,50,51,52,53]. The presence of dissolved oxygen is necessary for the oxidation and subsequent dissolution of silver that causes the toxic effect [54,55,56,57,58,59]. Therefore, we studied the release of silver ions from ultrasmall nanoparticles by filtration experiments through spin filters (3 kDa). The nanoparticles were incubated in water in closed vessels but without degassing at 4 °C, 25 °C, and 37 °C for up to 14 days and then separated from released silver ions by spin filtration. The filtrate was analyzed by AAS for silver ions. The procedure was validated with the analysis of a solution of silver nitrate (full permeation through the spin filter), a dispersion of gold nanoparticles (GSH-coated; 2 nm; no permeation through the spin filter), and as control a mixture of dissolved silver nitrate and dispersed gold nanoparticles. This gave the expected results, i.e., Ag^+^ ions went through the filter and gold nanoparticles were restrained. Only very small amounts of silver ions were detected for the Ag and Ag_50_Pt_50_ nanoparticles, irrespective of storage time and temperature (Figure 6). In other words, no significant dissolution of the nanoparticles was detected for monometallic and bimetallic silver nanoparticles.

## 4. Discussion

All nanoparticles had a uniform, mostly spherical shape and were well dispersible in water. Therefore, it is not surprising that they were easily taken up by HeLa cells. The nature of the metal core did not influence the uptake by cells or the intracellular localization; i.e., the interaction with cells was governed by their surface chemistry (e.g., the surface charge) [60]. The accumulation of the nanoparticles inside the cells suggests their presence inside endolysosomes [61]. However, the nanoparticles did not enter the cell nucleus (at least not to a significant extent), as sometimes reported for ultrasmall nanoparticles [35,62]. This is well in line with earlier observations on fluorescent ultrasmall nanoparticles. A size-dependent intracellular localization was reported for MCF7 breast cancer cells. Gold nanoparticles of 2 and 6 nm diameter were found in the nucleus, while their larger plasmonic analogues (10 and 16 nm) reached only the cytoplasm [62]. Yang et al. [63] and Carrillo-Carrion et al. [64] reported similar results for gold nanoparticles and quantum dots in the ultrasmall size range. Uptake studies of fluorescent ultrasmall gold nanoparticles (2 nm) in CT-26 cells to elucidate the uptake mechanism of ultrasmall nanoparticles were performed in the presence of various endocytosis inhibitors. It was shown that the uptake of the nanoparticles with CT-26 cells was not inhibited by a single endocytosis inhibitor. However, cooling to 4 °C led to a strong inhibition of the uptake. This excludes a purely diffusion-controlled migration across the cell membrane. Clearly, a combination of different endocytosis pathways is active for the uptake of ultrasmall nanoparticles [65].

Despite the considerable uptake by HeLa cells, we observed no significant toxic effect for ultrasmall gold and platinum nanoparticles, except at very high concentrations, well in line with their noble and inert nature. Ultrasmall gold nanoparticles were studied in depth regarding their cytotoxicity. For example, ultrasmall gold nanoparticles terminated with GSH (1.7 nm) were not cytotoxic for Hela cells up to 32 µg gold mL^−1^ [66]. Gold clusters were cytotoxic only below 2 nm size and only if the gold surface was accessible for biomolecules or cell constituents, which is the case for phosphane-ligand stabilized nanoparticles [67]. Rostek et al. prepared poly(N-vinylpyrrolidone) (PVP)-stabilized spherical gold, platinum, and silver nanoparticles with a size of 4 to 8 nm and incubated hMSC cells with them. Neither platinum nor gold nanoparticles had any effect on cell viability. Only silver nanoparticles showed a cytotoxic activity [68].

Surprisingly, monometallic ultrasmall silver nanoparticles showed a low cytotoxicity on HeLa cells and bacteria at all concentrations in our study. However, the effect was small as the release of silver ions was almost negligible. In general, the toxic concentration of silver is about 1–10 µg mL^−1^ for silver ions and 10–100 µg mL^−1^ for silver nanoparticles, depending on the cell type and the cultivation conditions [13]. For bacteria, it is of the order of 0.1 to 1 µg mL^−1^ for silver ions and 0.1 to 1 µg mL^−1^ for silver nanoparticles, depending on the bacterium, the culture conditions, and the type of nanoparticle [13]. For *Staphylococcus aureus*, larger silver nanoparticles of different size and shape (10 to 100 nm) were reported with a MIC of 25 to 50 µg mL^−1^ [69]. In a comparative study, the toxic effect of silver administered as ions (silver acetate) occurred at 0.5 to 5 µg mL^−1^ for *E. coli*, *S. aureus*, human mesenchymal stem cells (hMSCs), and peripheral blood mononuclear cells (PBMCs). PVP-stabilized silver nanoparticles (70 nm) gave a cytotoxic effect on the same bacteria and cells in the concentration range of 12.5 to 50 µg mL^−1^ for silver nanoparticles [14]. It was also demonstrated a decade ago that silver chloride is immediately formed after release of silver ions from silver nanoparticles [70], and that silver nanoparticles are neither toxic to bacteria [71] nor to cells [58,71] in the absence of oxygen, i.e., without oxidative dissolution. In contrast, ultrasmall GSH-coated silver nanoparticles (2 nm) showed a remarkably high cytotoxicity where about 50% of HeLa cells were dead at a concentration of 15 µg mL^−1^ after 3 h and at a concentration of 1 µg mL^−1^ after 24 h. These nanoparticles were also easily taken up by HeLa cells within 24 h and found in the cytosol [72]. The difference to our study is probably the fact that the particles analyzed in our study were stored for 4 weeks in dispersion for aging.

It shall be noted that all particle characterization data (physico-chemical and biological) that are reported here were obtained with nanoparticles that were immersed in water at ambient temperature for four weeks. During the initial experiments, it turned out that the cytotoxicity towards cells and bacteria varied with the age of the particles in an erratic way, although their physico-chemical properties like particle size did not significantly change. After four weeks of storage, all data were consistent and reproducible. It is possible that the particles underwent an internal change during immersion in water that influenced their internal structure and their biological effects. This would also explain the much higher cytotoxicity observed for freshly prepared silver nanoparticles [72]. However, the elucidation of this effect would require extensive time-resolved studies that were beyond the scope of this study.

The cytotoxicity of the bimetallic silver-platinum nanoparticles was higher than that of monometallic silver nanoparticles, both for HeLa cells and bacteria. The critical concentrations for a 50% cytotoxicity for HeLa cells were about 50 µg mL^−1^ for Ag_70_Pt_30_ and Ag_50_Pt_50_ and 75 µg mL^−1^ for the physical mixture of Ag/Pt nanoparticles. A 50% cytotoxicity was not reached for Au, Ag, Pt, and Ag_20_Pt_80_ nanoparticles. Notably, Ag_50_Pt_50_ nanoparticles were significantly more cytotoxic than an equimolar physical mixture (50:50) of Ag and Pt nanoparticles [73]. The MIC values of bimetallic nanoparticles were close to or even lower than for silver nitrate, used in this study as reference and source of bactericidal silver ions. Thus, the bimetallic silver-platinum nanoparticles were more toxic against HeLa cells and bacteria than monometallic silver nanoparticles. This cannot be due to an enhanced release of silver ions as the bimetallic Ag_50_Pt_50_ nanoparticles did not dissolve in water.

Many of the available reports on studies on the antibacterial activity of bimetallic nanoparticles compared to their monometallic counterparts are based on the application of Au-Ag nanoparticles, but the mechanism of their bactericidal action remains not fully understood [74]. Little is known about the cytotoxicity of bimetallic silver–platinum nanoparticles so far. There is an ongoing discussion on a sacrificial anode effect in alloyed nanoparticles which could lead to polarization and an enhanced dissolution of cytotoxic silver ions. It has been shown theoretically that a noble metal can protect a less noble metal from oxidation [75]. On the other hand, a noble metal can enhance the dissolution rate by increasing the apparent charge of Ag^0^ to Ag^+^ by electrochemical polarization [76]. A potential sacrificial anode effect was reported for silver–platinum surface coatings [77,78,79,80]. Grasmik et al. prepared bimetallic AgPt nanoparticles of 15 to 25 nm diameter with PVP coating and found a cytotoxicity against hMSC above a silver content of 50 mol%, supported by silver-release experiments where silver was only released above 50 mol% silver [22]. Breisch et al. studied the antibacterial effects of a physical mixture of PVP-coated Ag and Pt nanoparticles (both 7 nm) against *E. coli* and *S. aureus* and observed an enhancement of the cytotoxicity of silver nanoparticles in the presence of platinum nanoparticles [73]. In contrast, alloyed nanoparticles (10 nm; PVP-coated) of the compositions Ag, Ag_10_Pt_90_, Ag_30_Pt_70_, Ag_50_Pt_50_, Ag_70_Pt_30_, Ag_90_Pt_10_, and Pt did not show an enhanced bactericidal effect [21]. Yang et al. prepared 2.1 nm bimetallic nanoparticles of Ag and Cu and reported a considerably enhanced silver release rate [81]. Singh et al. prepared bovine serum albumin (BSA)-capped bimetallic AgPt nanoparticles of 10 to 15 nm diameter but did not find an adverse effect on the viability and the morphology of human gingival fibroblasts exposed to the nanoparticles for 24 h. This was in sharp contrast to the precursor salts that released silver or platinum ions, i.e., Ag_2_SO_4_ and H_2_PtCl_6_, respectively, suggesting that the surface of the nanoparticles was well blocked by attached thiol groups from the BSA. However, the authors did not report the Ag:Pt ratio in their particles [82].

Regardless of the presence or absence of a sacrificial anode effect, the cytotoxicity of silver-containing nanoparticles has always been related to the release of silver ions [11,13,83,84]. In most studies, the silver-containing nanoparticles were surface-stabilized by ligands with low surface affinity, e.g., PVP. The low cytotoxicity of GSH-stabilized silver nanoparticles indicates that the surface ligand plays a decisive role in the cytotoxicity. The observed very low dissolution rate of GSH-coated silver nanoparticles is in line with earlier results where PVP-coated silver nanoparticles (70 nm) did not show a release of silver ions in the presence of cysteine [55]. Cysteine obviously blocked the surface of the metal nanoparticles by firm adsorption, replacing the initial ligand PVP. This was also found by Liu et al. and Levard et al., who reviewed different approaches to control the release of silver ions from silver nanoparticles. They concluded that the release of silver ions is restricted by sulfidation or by thiol ligands on the silver nanoparticle surface [57,85,86,87]. It has been shown by various methods that the sulfur–silver bond is very strong [30,72,88,89] and apparently prevents the release of silver ions by oxidation. It should also be considered that the ligand density on the surface of an ultrasmall nanoparticle is very high (14 to 29 GSH units per nm^2^); i.e., the metal surface is probably well protected against oxidative attack from dissolved oxygen. This is obviously also the case for ultrasmall nanoparticles with their very high specific surface area. The oxidized state of the particles as shown by XPS for both silver and for platinum implies the presence of oxidized surface species, probably together with glutathione via the sulfur atom. However, it must be cautioned that the dissolution in water may be different from that in cell culture media where a complexation of silver ions by proteins or other biomolecules is well conceivable. Unfortunately, it is not possible to study the dissolution of ultrasmall silver nanoparticles in biological media like RPMI-FCS because particles and ions cannot be separated in the presence of high protein concentrations (nanoparticles and proteins are of the same dimension and cannot be separated by spin filtration) [24].

It is remarkable that the highest cytotoxicity against eukaryotic cells and bacteria was observed for the bimetallic nanoparticles, especially with an equimolar ratio of silver to platinum. They were much more cytotoxic than a physical mixture of the monometallic nanoparticles. This cannot be ascribed to the thiolated surface which is also present in monometallic silver nanoparticles. Although the exact mechanism of the cytotoxic action is not known, it is likely that the oxidized nature of the metals (especially of silver) influences the biological effects, possibly after cellular uptake.

## 5. Conclusions

All types of ultrasmall nanoparticles are easily taken up by eukaryotic cells (HeLa), regardless of the composition of their metal core. Bimetallic nanoparticles have a higher cytotoxicity and a stronger bactericidal effect than a physical mixture of silver and platinum nanoparticles and the monometallic nanoparticles, both against HeLa cells and bacteria (*E. coli*, Gram-negative, and *S. xylosus*, Gram-positive). This is not due to an enhanced dissolution, e.g., by an oxidative release of silver ions. The very dense surface coverage by the strongly attached ligand glutathione (bound by the strong Ag-S bond) is probably responsible for the very low release of silver ions. Nevertheless, the fact that the bimetallic nanoparticles are consistently more cytotoxic than the monometallic silver nanoparticles, although both do not release silver ions upon immersion in water, must be due their bimetallic nature. Obviously, the bimetallic AgPt nanoparticles exert an additional cytotoxic effect which occurs only in biological media and cannot be explained by the ion release in water. It is well conceivable that an enhanced dissolution mediated by biomolecules and increased by polarization of silver by platinum is responsible for this effect.

## Figures and Tables

**Table 1:** Reaction conditions for the synthesis of N_3_-terminated nanoparticles (M-N_3_). The nanoparticle concentration was computed assuming spherical nanoparticles of 2 nm diameter. All other synthesis conditions were the same as with gold nanoparticles. The yield was 50 to 90% with respect to the total metal content.

	Au	Ag	Ag_50_Pt_50_	Pt
GSH-terminated nanoparticles used for the synthesis	15 mg Au, 0.31 µmol nanoparticles	15 mg Ag, 0.57 µmol nanoparticles	6.47 mg Ag, 11.7 mg Pt, 0.48 µmol nanoparticles	18.8 mg Pt, 0.35 µmol nanoparticles
round-bottomed flask	250 mL	500 mL	250 mL	250 mL
H_2_O	18 mL	36 mL	20 mL	24 mL
methanol	54 mL	108 mL	60 mL	72 mL
ISA	184 mg, 676 µmol	394 mg, 1.45 mmol	221 mg, 812 µmol	357 mg, 1.31 mmol
K_2_CO_3_	94 mg, 676 µmol	201 mg, 1.45 mmol	113 mg, 812 µmol	183 mg, 1.31 mmol
5 mM CuSO_4_ solution	1.04 mL, 5.2 µmol	2.52 mL, 12.6 µmol	3.67 mL, 18.4 µmol	3.91 mL, 19.6 µmol
1 M NaOH solution	1 mL	2 mL	2 mL	4 mL
Reaction time	72 h	48 h	48 h	72 h

**Table 2:** Reaction conditions for the synthesis of AF647-terminated nanoparticles (M-AF647). The nanoparticle concentration was computed assuming spherical nanoparticles of 2 nm diameter. All other synthesis conditions were the same as with gold nanoparticles. The yield was about 80% with respect to the total metal content.

	Au	Ag	Ag_50_Pt_50_	Pt
N_3_-terminated nanoparticles used for the synthesis	3 mg Au, 62 nmol nanoparticles	2.54 mg Ag, 96 nmol nanoparticles	1.85 mg Pt, 0.61 mg Ag, 61 nmol nanoparticles	3.7 mg Pt, 68 nmol nanoparticles
11.2 mM AF647-alkyne solution	100 µL, 1.12 µmol, 1 mg	155 µL, 1.73 µmol, 1.54 mg	100 µL, 1.12 µmol, 1 mg	61 µL, 0.68 µmol, 0.61 mg
Cu-THPTA solution	583 µL	853 µL	459 µL	1.5 mL
10 mM sodium ascorbate solution	301 µL, 3.6 µmol, 0.6 mg	246 µL, 2.46 µmol, 0.49 mg	164 µL, 1.6 µmol, 0.33 mg	535 µL, 5.4 µmol, 1.06 mg
Reaction time	17 h	6 h	17 h	17 h

**Table 3:** Analytical data of all prepared and investigated GSH-coated ultrasmall nanoparticles.

	Ag	Ag_70_Pt_30_	Ag_50_Pt_50_	Ag_20_Pt_80_	Pt	Au
particle core volume/nm^3^	4.19 *	4.19 *	4.19 *	4.19 *	4.19 *	4.19 *
particle core weight/g·10^23^	4.39 *	5.54 *	6.69 *	7.84 *	8.98 *	8.09 *
particle density/g cm^−3^	10.49	13.78 **	15.97 **	19.26 **	21.45	19.32
particle core surface area/nm^2^	12.57 *	12.57 *	12.57 *	12.57 *	12.57 *	12.57 *
hydrodynamic diameter (DCS)/nm	1.7 ± 0.5 ***	1.5 ± 0.3	1.6 ± 0.3 ***	1.6 ± 0.4	1.6 ± 0.4 ***	1.5 ± 0.3 ***
diffusion coefficient (^1^H-DOSY)/10^−10^ m^2^ s^−1^	1.47 ***	1.53	1.60 ***	1.69	1.56 ***	1.28 ***
hydrodynamic diameter (^1^H-DOSY)/nm	3.32 ***	3.19	3.05 ***	2.88	3.14 ***	3.81 ***
particle core diameter (HRTEM)/nm	2.2 ± 0.5 ***	1.9 ± 0.6	1.8 ± 0.4 ***	1.8 ± 0.3	2.0 ± 0.4 ***	2.0 ± 0.4 ***
particle core diameter (SAXS)/nm	1.0 ± 0.1 ***	1.0 ± 0.1	1.6 ± 0.1 ***	1.0 ± 0.1	0.9 ± 0.1 ***	0.8 ± 0.2
crystallinity by TEM	crystalline ***	amorphous	amorphous ***	crystalline	crystalline ***	crystalline ***
oxidation state of metals by XPS	Ag^+^ ***	Ag^+^, Pt, Pt^2+^	Ag^+^, Pt, Pt^2+^ ***	Ag^+^, Pt, Pt^2+^	Pt, Pt^2+^ ***	Au ***
normalized molar ratio metal(M):sulfur(S) by ICP-MS	1.00 (Ag):1.28 (S) ***	0.71 (Ag):0.29 (Pt):0.65 (S)	0.59 (Ag):0.41 (Pt):0.68 (S) ***	0.16 (Ag):0.84 (Pt):1.35 (S)	1.00 (Pt):0.73 (S) ***	1.00 (Au):0.82 (S) ***
overall nominal composition of one nanoparticle	Ag_245_GSH_315_ ***	Ag_184_Pt_74_GSH_170_	Ag_156_Pt_110_GSH_180_ ***	Ag_43_Pt_230_GSH_370_	Pt_277_GSH_206_ ***	Au_247_GSH_203_ ***
GSH molecular footprint/nm^2^	0.040 ***	0.074	0.070 ***	0.034	0.062 ***	0.062 ***
number of conjugated AlexaFluor-647 molecules on each M-AF647 nanoparticle by AAS and UV-VIS	13	-	8	-	6	12

*: Computed values based on the assumption that the average particle diameter was 2 nm and that the nanoparticles were spherical. ** Computed from the stoichiometry as determined with ICP-MS. *** The data of GSH-coated Ag, Au, Pt, and Ag_50_Pt_50_ nanoparticles were taken from ref. [30].

**Table 4:** Cell viabilities (in %) by the MTT test of GSH-coated nanoparticles as function of the total metal content *c*(metal) (mean ± standard deviation in percent). For silver-containing particles, the effective silver concentration is given in parentheses in µg mL^−1^.

*c*(Metal)/in µg mL^−1^	Au	Ag	Ag_70_Pt_30_	Ag_50_Pt_50_	Ag/Pt (50:50)	Ag_20_Pt_80_	Pt
0	100 ± 6	100 ± 5 (Ag: 0)	100 ± 4 (Ag: 0)	100 ± 9 (Ag: 0)	100 ± 4 (Ag: 0)	100 ± 2 (Ag: 0)	100 ± 10
2.5	93 ± 6	92 ± 6 (Ag: 2.5)	105 ± 2 (Ag: 1)	107 ± 2 (Ag: 0.9)	99 ± 7 (Ag: 0.9)	98 ± 4 (Ag: 0)	100 ± 3
5	99 ± 4	88 ± 7 (Ag: 5)	110 ± 1 (Ag: 2.8)	114 ± 4 (Ag: 1.8)	100 ± 6 (Ag: 1.8)	113 ± 5 (Ag: 0.6)	97 ± 8
10	105 ± 5	79 ± 6 (Ag: 10)	95 ± 4 (Ag: 5.6)	99 ± 4 (Ag: 3.6)	71 ± 12 (Ag: 3.6)	99 ± 10 (Ag: 1)	90 ± 5
15	93 ± 4	77 ± 5 (Ag: 15)	90 ± 5 (Ag: 8.5)	94 ± 5 (Ag: 5)	62 ± 12 (Ag: 5)	99 ± 4 (Ag: 1.8)	84 ± 5
25	102 ± 6	91 ± 9 (Ag: 25)	84 ± 6 (Ag: 14)	97 ± 5 (Ag: 8.9)	60 ± 8 (Ag: 8.9)	105 ± 6 (Ag: 3)	91 ± 4
50	93 ± 11	89 ± 10 (Ag: 50)	26 ± 10 (Ag: 28)	47 ± 6 (Ag: 18)	54 ± 7 (Ag: 18)	100 ± 4 (Ag: 6)	90 ± 8
75	91 ± 6	78 ± 4 (Ag: 75)	15 ± 10 (Ag: 42)	13 ± 3 (Ag: 27)	46 ± 14 (Ag: 27)	85 ± 6 (Ag: 9)	85 ± 3
100	97 ± 4	76 ± 5 (Ag: 100)	11 ± 5 (Ag: 56)	8 ± 4 (Ag: 36)	44 ± 14 (Ag: 36)	69 ± 3 (Ag: 12)	87 ± 7

**Table 5:** MIC values determined for *E. coli* DH5α and *S. xylosus* DSM 6179 after 24 h of incubation with different doses of GSH-coated nanoparticles, given in µg mL^−1^ (metal content). The effective silver concentration is given in parentheses in µg mL^−1^, where appropriate. *E*. *coli* (CFU: 1.1·10^8^ cells mL^−1^; cell dose: 1.1·10^6^ cells per well), *S*. *xylosus* (CFU: 5·10^7^ cells mL^−1^; cell dose: 5·10^5^ cells per well).

Sample	*E. coli* (Gram-Negative)	*S. xylosus* (Gram-Positive)
AgNO_3_	6–10 (6–10)	15–25 (15–25)
Ag	>100 (>100)	>100 (>100)
Au	>100 (−)	>100 (−)
Pt	>100 (−)	>100 (−)
Ag_50_Pt_50_	11–15 (4–5.4)	1–5 (0.4–1.8)
Ag/Pt (50:50)	76–100 (27–36)	>100 (>36)

## Data Availability

The original contributions presented in the study are included in the article/Supplementary Material; further inquiries can be directed to the corresponding author.

## Supplementary Materials

The following supporting information can be downloaded at: https://www.mdpi.com/article/10.3390/ma17153702/s1: Figure S1: Differential centrifugal sedimentation (DCS) of bimetallic Ag_70_Pt_30_ and Ag_20_Pt_80_ nanoparticles; Figure S2: SAXS on bimetallic Ag_70_Pt_30_ and Ag_20_Pt_80_ nanoparticles; Figure S3: X-ray powder diffraction on bimetallic Ag_70_Pt_30_ and Ag_20_Pt_80_ nanoparticles; Figure S4: XPS measurements on bimetallic Ag_70_Pt_30_ and Ag_20_Pt_80_ nanoparticles; Figure S5: ^1^H NMR spectra of Ag_70_Pt_30_ and Ag_20_Pt_80_ nanoparticles; Figure S6: ^13^C-DEPTQ NMR spectra of Ag_70_Pt_30_ and Ag_20_Pt_80_ nanoparticles; Figure S7: ^1^H-^1^H COSY NMR spectra of Ag_70_Pt_30_ and Ag_20_Pt_80_ nanoparticles; Figure S8: ^1^H-^13^C HSQC NMR spectra of Ag_70_Pt_30_ and Ag_20_Pt_80_ nanoparticles; Figure S9: ^1^H-^13^C-HMBC NMR spectra of Ag_70_Pt_30_ and Ag_20_Pt_80_ nanoparticles; Figure S10: IR spectra of GSH- und N_3_-terminated gold, silver, platinum, and bimetallic silver–platinum nanoparticles.
