# Supplementary material for: Increased Cytotoxicity of Bimetallic Ultrasmall Silver–Platinum Nanoparticles (2 nm) on Cells and Bacteria in Comparison to Silver Nanoparticles of the Same Size
Source: Materials (Basel). 2024 Jul 26;17(15):3702. doi: 10.3390/ma17153702 (PMC11313250; doi:10.3390/ma17153702)
Supplement: Supplementary file 1 [file materials-17-03702-s001.zip › materials-3121523-supplementary.pdf]

Supplementary Information

Increased cytotoxicity of bimetallic ultrasmall silver-platinum nanoparticles (2 nm) on cells and bacteria in comparison to silver nanoparticles of the same size

Natalie Wolff,[1] Nataniel Białas,[1] Kateryna Loza,[1] Marc Heggen,[2] Torsten Schaller,[3] Felix Niemeyer,[3] Claudia Weidenthaler,[4] Christine Beuck,[5] Peter Bayer,[5] Oleg Prymak,[1] Cristiano L. P. Oliveira,[6] Matthias Epple[1,*]

[1] Inorganic Chemistry and Centre of Nanointegration Duisburg-Essen (CENIDE), University of Duisburg-Essen, Universitaetsstr. 5-7, 45117 Essen, Germany

[2] Ernst Ruska Centre for Microscopy and Spectroscopy with Electrons, Forschungszentrum Jülich, 52428 Jülich, Germany

[3] Organic Chemistry, University of Duisburg-Essen, Universitaetsstr. 5-7, 45117 Essen, Germany

[4] Max-Planck-Institut für Kohlenforschung, Mülheim an der Ruhr 45470, Germany

[5] Institute of Biology and Center for Medical Biotechnology (ZMB), University of Duisburg-Essen, Universitaetsstr. 5-7, 45117 Essen, Germany

[6] Institute of Physics, University of São Paulo, São Paulo 05508-090, Brazil

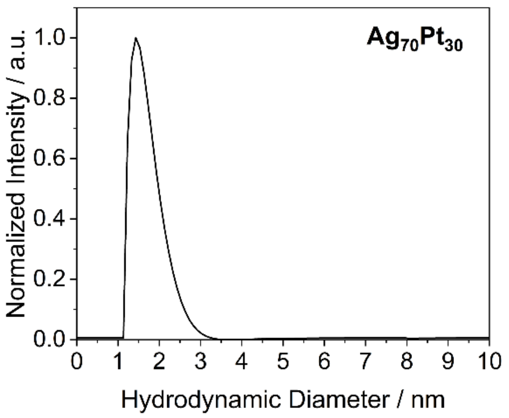

Figure S1: Differential centrifugal sedimentation (DCS) of bimetallic GSH-coated $Ag_{70}Pt_{30}$ and $Ag_{20}Pt_{80}$ nanoparticles.

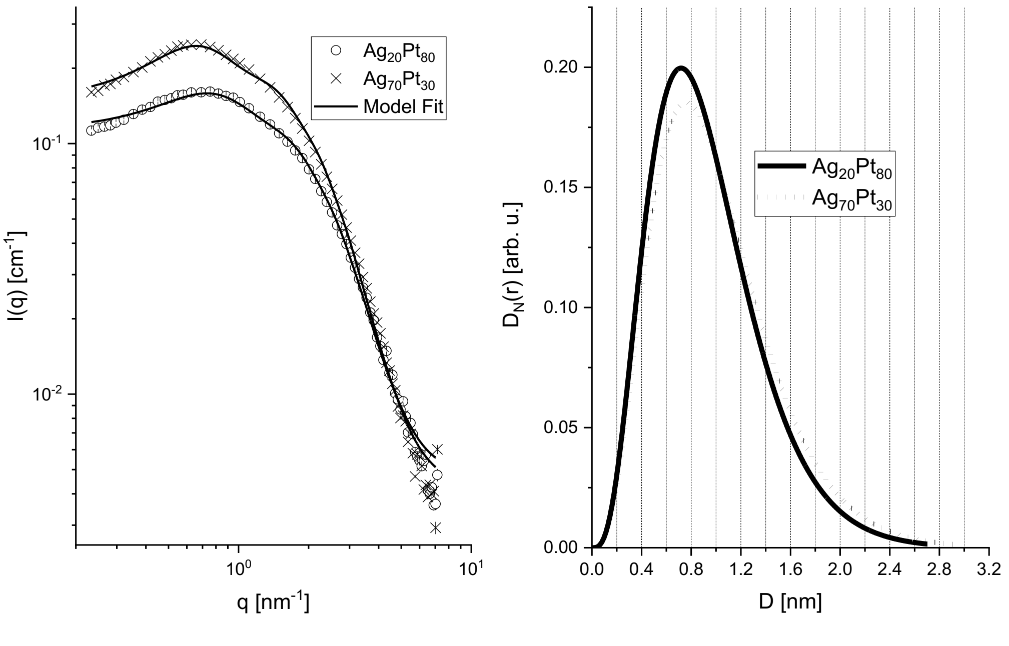

Figure S2: Small-angle X-ray scattering (SAXS) on bimetallic GSH-coated $Ag_{70}Pt_{30}$ and $Ag_{20}Pt_{80}$ nanoparticles.

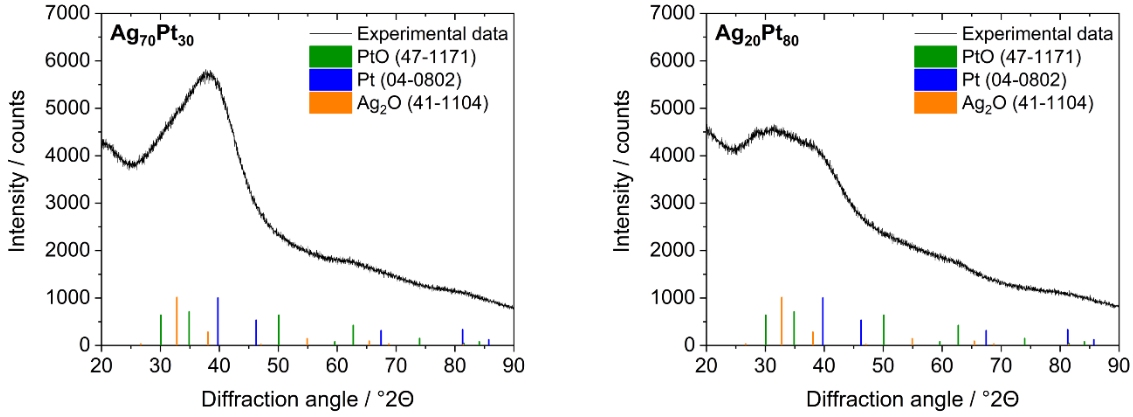

Figure S3: X-ray powder diffraction on bimetallic GSH-coated $Ag_{70}Pt_{30}$ and $Ag_{20}Pt_{80}$ nanoparticles.

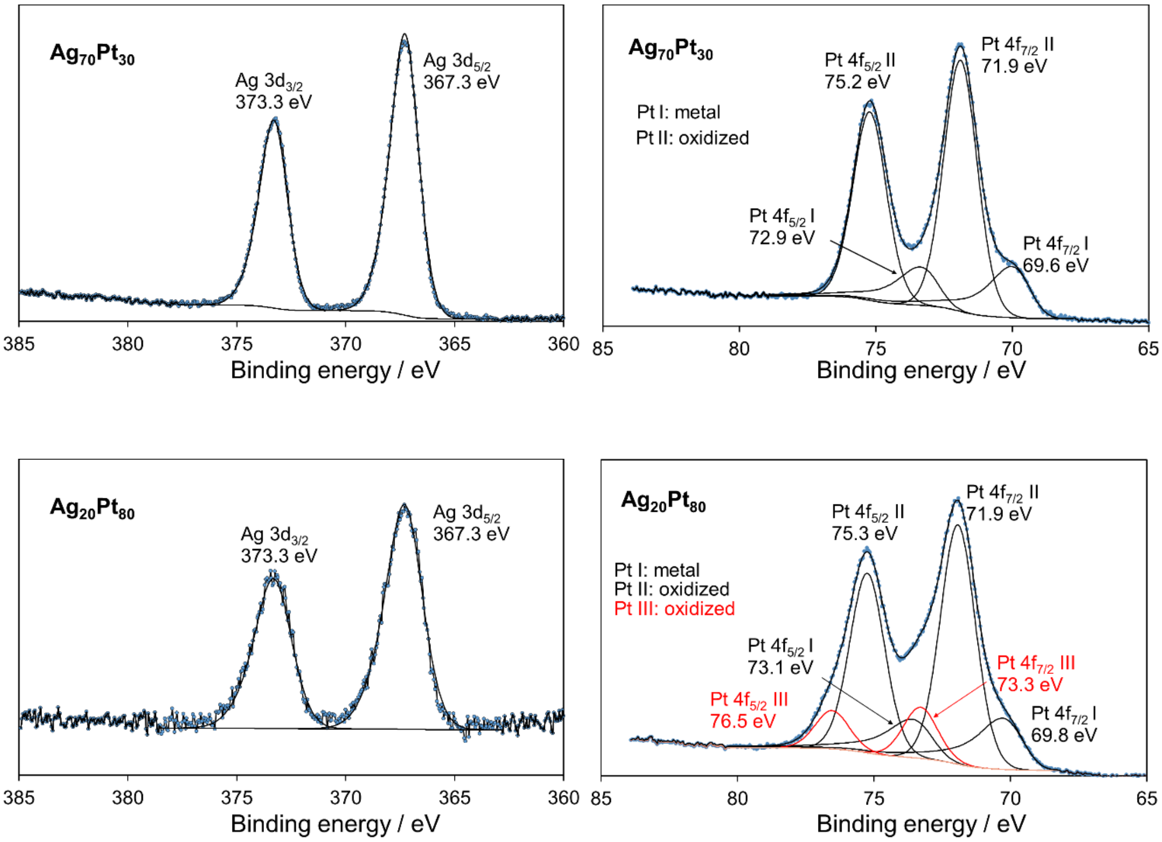

Figure S4: XPS core level spectra of Ag 3d and Pt 4f obtained for GSH-coated $Ag_{20}Pt_{80}$ and $Ag_{70}Pt_{30}$ nanoparticles. Silver is present as Ag^{+}, and platinum is present as Pt^{0} and Pt^{2+}.

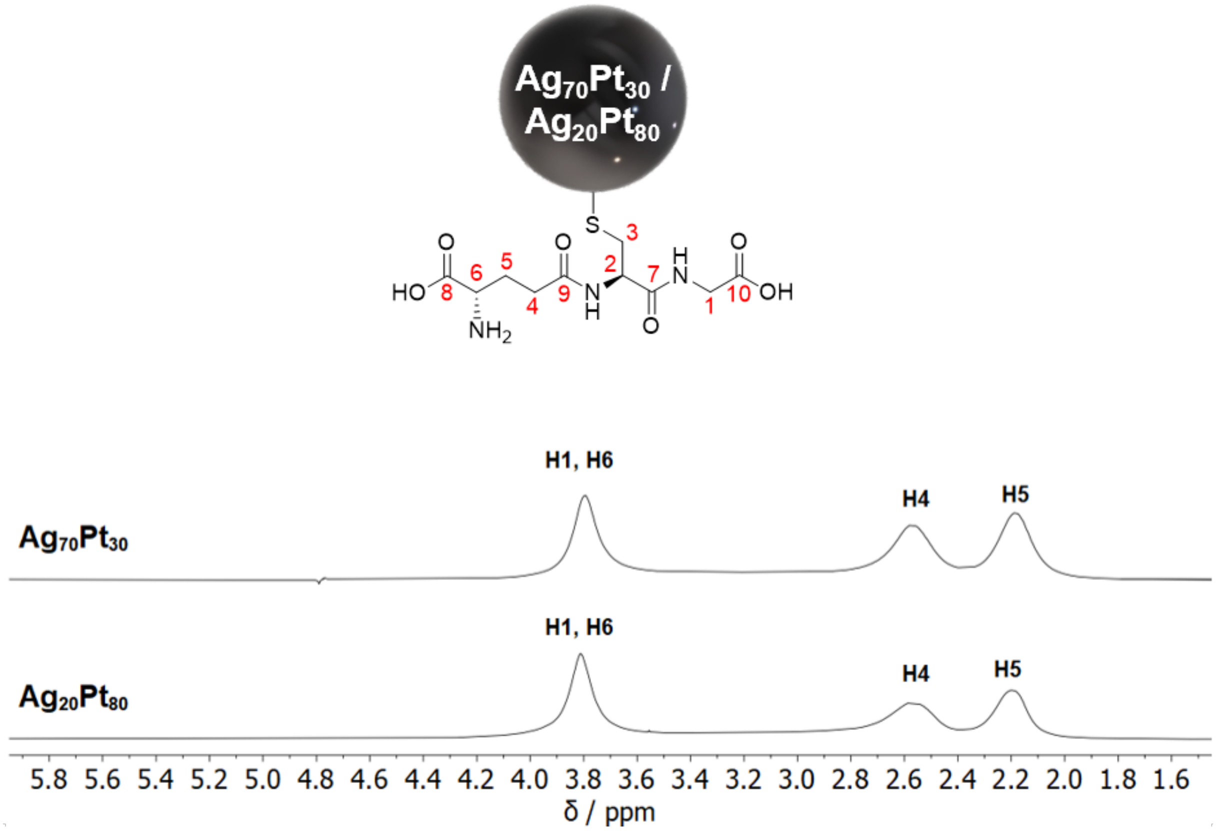

Figure S5: ^{1}H NMR spectra of bimetallic GSH-coated $Ag_{70}Pt_{30}$ and $Ag_{20}Pt_{80}$ nanoparticles (100% D_2O; pH 8.5). The strongly broadened NMR signals are due to the platinum-containing core of the nanoparticles.

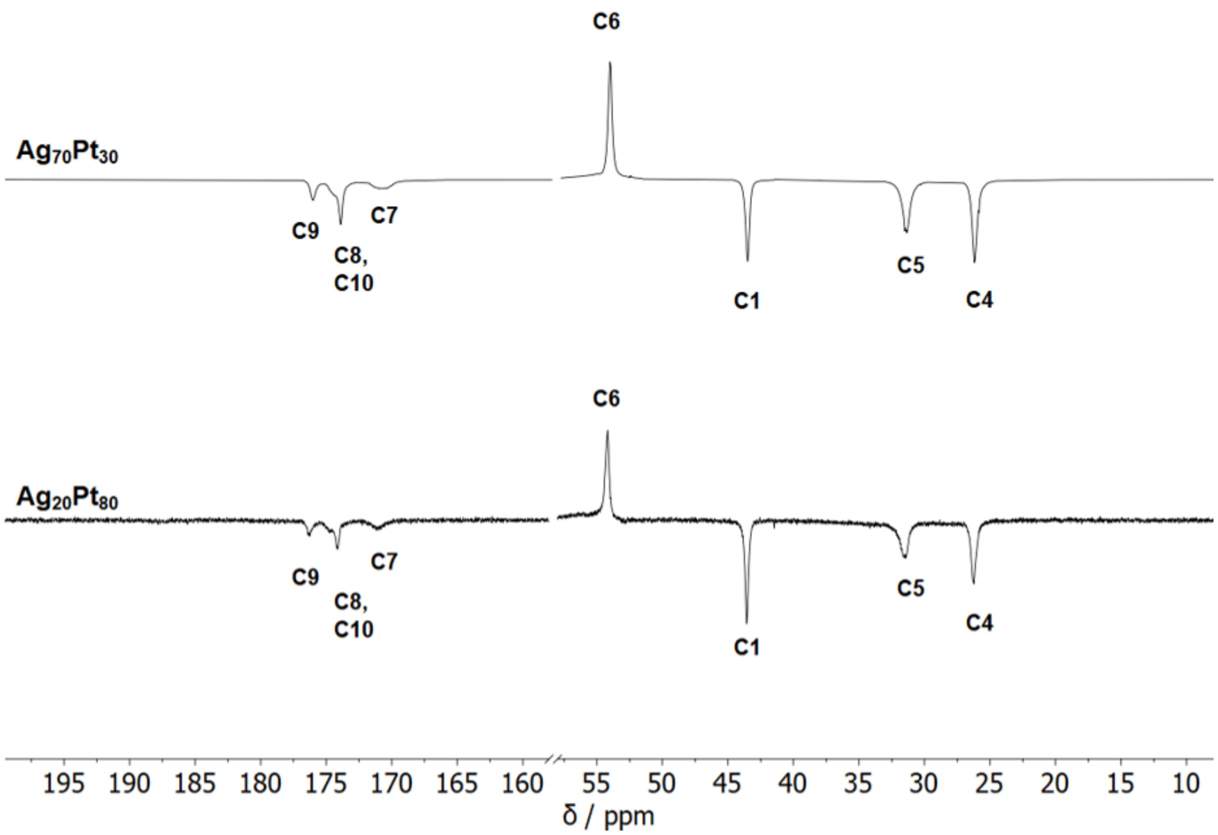

Figure S6: ^{13}C-DEPTQ NMR spectra of bimetallic GSH-coated $Ag_{70}Pt_{30}$ and $Ag_{20}Pt_{80}$ nanoparticles (100% D_2O; pH 8.5). The strongly broadened NMR signals are due to the platinum-containing core of the nanoparticles.

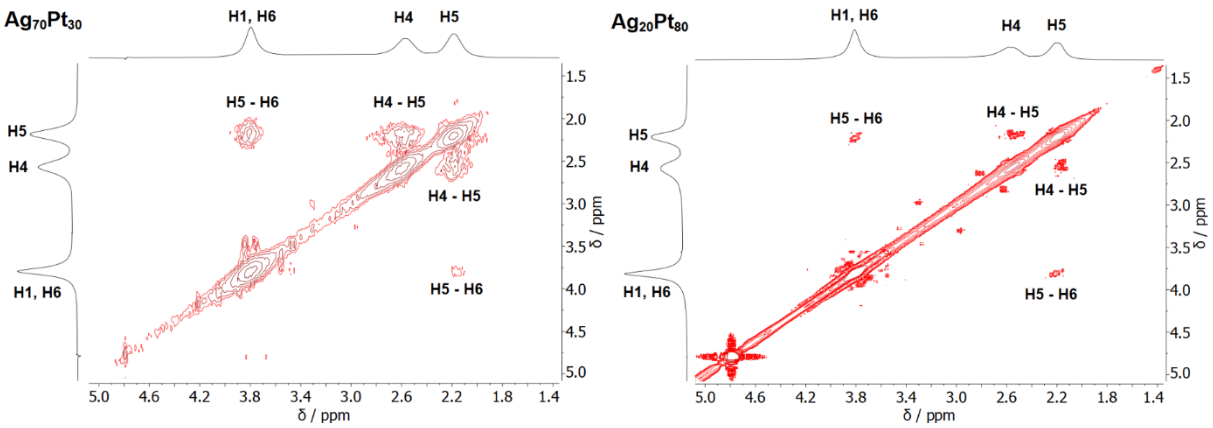

Figure S7: ^{1}H-^{1}H COSY NMR spectra of bimetallic GSH-coated $Ag_{70}Pt_{30}$ and $Ag_{20}Pt_{80}$ nanoparticles (100% D_2O; pH 8.5).

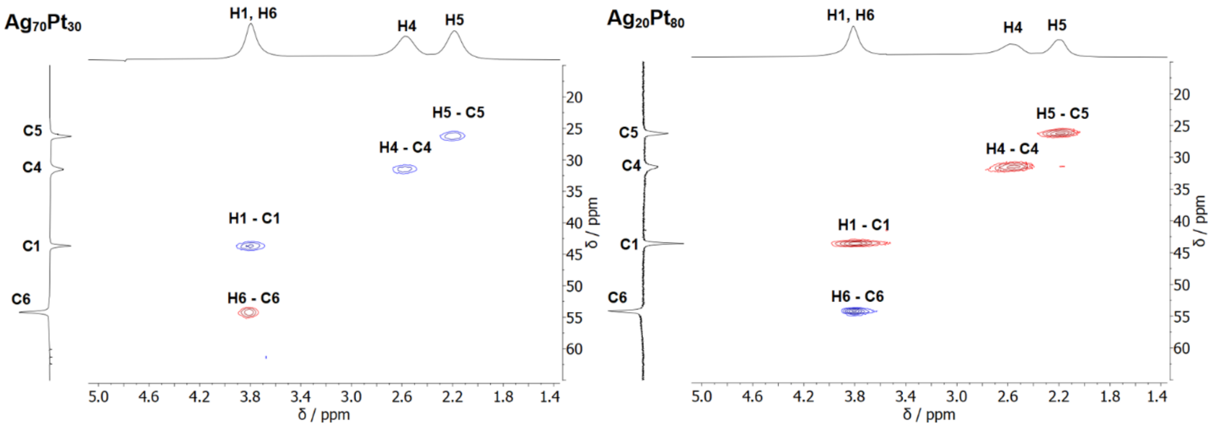

Figure S8: ^{1}H-^{13}C HSQC NMR spectra of bimetallic GSH-coated $Ag_{70}Pt_{30}$ and $Ag_{20}Pt_{80}$ nanoparticles (100% D_2O; pH 8.5). CH_2 and CH/CH_3 carbon atoms can be distinguished by this method (labelled in different colors).

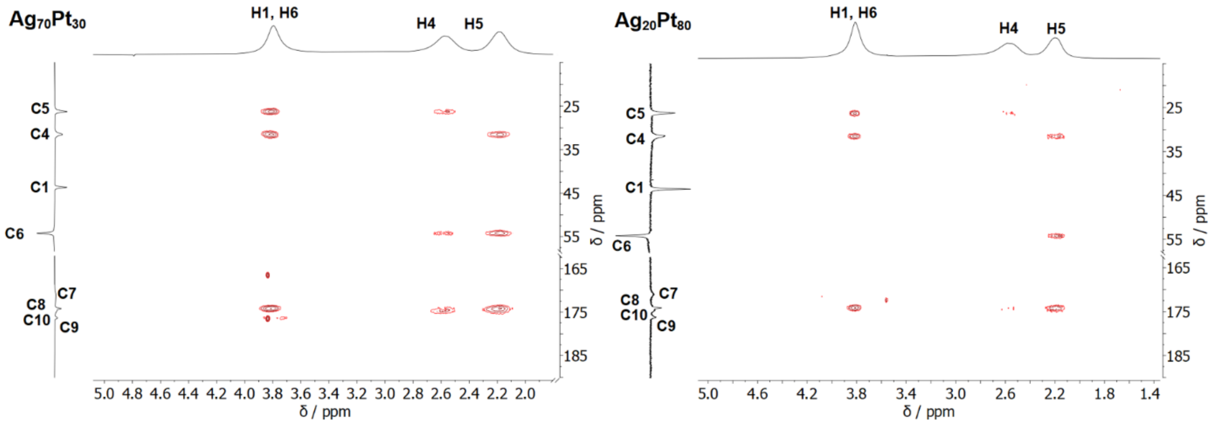

Figure S9: ^{1}H-^{13}C-HMBC NMR spectra of bimetallic GSH-coated $Ag_{70}Pt_{30}$ and $Ag_{20}Pt_{80}$ nanoparticles (100% D_2O; pH 8.5).

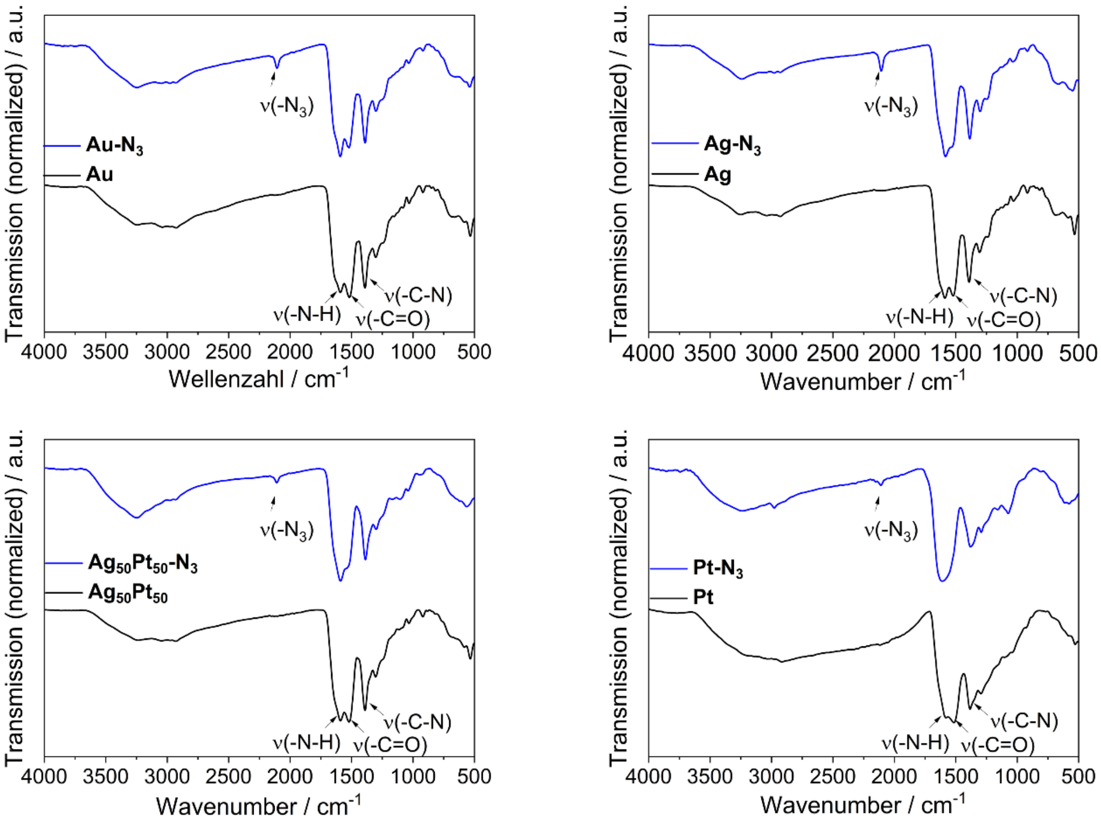

Figure S10: IR spectra of GSH- und N_3-terminated gold, silver, platinum and bimetallic silver-platinum nanoparticles. The azide band at 2106 cm^{-1} indicates the successful surface modification of all nanoparticles.
